# Microfluidic active pressure and flow stabiliser

**DOI:** 10.1038/s41598-021-01865-4

**Published:** 2021-11-18

**Authors:** Simon Södergren, Karolina Svensson, Klas Hjort

**Affiliations:** Microsystems Technology Division, Centre of Natural Hazard and Disaster Science (CNDS), Uppsala University, Box 35, 751 03 Uppsala, Sweden

**Keywords:** Chemical engineering, Actuators, Fluidics, Techniques and instrumentation

## Abstract

In microfluidics, a well-known challenge is to obtain reproducible results, often constrained by unstable pressures or flow rates. Today, there are existing stabilisers made for low-pressure microfluidics or high-pressure macrofluidics, often consisting of passive membranes, which cannot stabilise long-term fluctuations. In this work, a novel stabilisation method that is able to handle high pressures in microfluidics is presented. It is based on upstream flow capacitance and thermal control of the fluid’s viscosity through a PID controlled restrictor-chip. The stabiliser consists of a high-pressure-resistant microfluidic glass chip with integrated thin films, used for resistive heating. Thereby, the stabiliser has no moving parts. The quality of the stabilisation was evaluated with an ISCO pump, an HPLC pump, and a Harvard pump. The stability was greatly improved for all three pumps, with the ISCO reaching the highest relative precision of 0.035% and the best accuracy of 8.0 ppm. Poor accuracy of a pump was compensated for in the control algorithm, as it otherwise reduced the capacity to stabilise longer times. As the dead volume of the stabiliser was only 16 nL, it can be integrated into micro-total-analysis- or other lab-on-a-chip-systems. By this work, a new approach to improve the control of microfluidic systems has been achieved.

## Introduction

A pressure or flow stabiliser is a device that reduces fluctuations (low frequency) and noise (high frequency) in a fluidic system. It is vital in both micro and macro scale fluidics to provide reliable and reproducible results. Some commonly used pumps, driven by dual pistons or step motors, are known for having a pulsatile behaviour with large fluctuations. In demanding applications and for the highest stability, also high pressure syringe pumps or pumps with already integrated dampers suffer from too high noise or long-term fluctuations. Examples requiring stable flow include high pressure synthesis^1^, precise extractions^2^, droplet formation^3^, and more reproducible separation and sensitive detection in chromatography^4–6^.

For pumps that show the correct pressure level, there can still be components like filters and tubes with varying restrictions, changing the pressure before it reaches the experiment. Further, for pumps with flow rate control, long stabilisation times cause gradients of the flow rate through the experiment. Using stabilisers and sensors directly connected to the experiment are therefore important for precise control.

Today, there are different types of stabilisers available. To reduce the noise, passive systems use either a flexible channel or membrane, or a non-flexible restrictor. To also control the fluctuations, active systems can use electro-pneumatics. There are several reported solutions for different applications in, e.g., low-pressure^3,7–18^ and high-pressure^19^ liquid systems, high-pressure gas systems, and as HPLC pulse dampers^20,21^.

However, the active systems suffer from large volumes while the smaller, passive, restrictors cannot handle fluctuations but only noise. Hence, there is a need for an active microfluidic high-pressure stabiliser, a device that has not yet been presented.

The objective of this work is to present a concept for active pressure and flow stabilisation by the use of a thermally actuated restrictor, based on the change of viscosity with temperature, and the upstream flow capacitance. An active microfluidic stabiliser with an extremely low dead volume has been developed, with usability in a wide range of applications. The low volume makes only a fraction of the fluid exposed for the temperature changes, which are quickly neutralised, leaving the temperature downstream of the stabiliser unaffected. The concept of the thermally actuated restrictor is demonstrated, and the performance of the stabilisation is evaluated with three commonly used pumps. The device consists of a chemically inert microfluidic glass chip with integrated thin-film heaters. The flow is regulated by changing the power through the heaters, which thermally adjusts the viscosity of the fluid, and thereby the restriction. With a PID control and an external sensor downstream, a feedback loop was made to control the heating in relation to the desired pressure or flow rate. The stabiliser uses the same actuation principle as the recently published active flow control board^22^ and a back pressure regulator^23^, but is making use of the compressibility of the fluid and its resulting flow capacitance, which has previously been an obstacle.

## Theory

To reduce fluctuations from a pump there has to be room for a buffer capacitance, meaning that the flow capacitance can both increase and decrease. The buffer capacitance enables fluid to be released when the pressure is too low, and stored when the pressure is too high. Since the flow rate and pressure are linearly correlated to each other by the Hagen Poiseuille equation, the same theory goes for stabilising the flow rate as for the pressure, Eq. (1)

1 $$\Delta P=\frac{8\mu LQ}{\pi {\left(\frac{{D}_{H}}{2}\right)}^{4}}$$

Here, one can observe how the pressure drop, *ΔP*, or the flow rate, *Q*, is also linearly dependent of the viscosity, *µ*, for a certain channel length, *L*, and hydraulic diameter, *D*_*H*_.

For dampers using a flexible membrane, there is a compressible gas on the opposite side of the fluid. When pressure rises, the membrane dilates and compresses the gas. In this way the volume of the fluid increases, which lowers the pressure. If the pressure gets too low, the membrane moves back while the gas decompresses. The buffer capacitance depends on the volume change the flexible membrane causes. At lower pressures, the same principle can be temporarily used with just an air bubble that compresses without any membrane^17^. Both cases use gas as the compressible media but there are also examples using moving membranes with liquids on both sides^24^.

However, by using a sufficient restriction, it is possible to stabilise the flow using only the compressibility of the fluid itself, instead of changing the volume. Though many liquids are referred to as incompressible, they never fully are. Water has normally a compressibility of 46 ppm/bar, which corresponds to a volume change of 4.6 µL/bar for a pump with a volume of 100 mL. In general, the effect of compressibility is mostly noticed in the long waiting time it causes. When the flow rate or pressure is changed, extra fluid has to be added or removed before it is stable, limiting several applications. Nonetheless, it can also be useful by reducing fluctuations.

**Figure 1:**
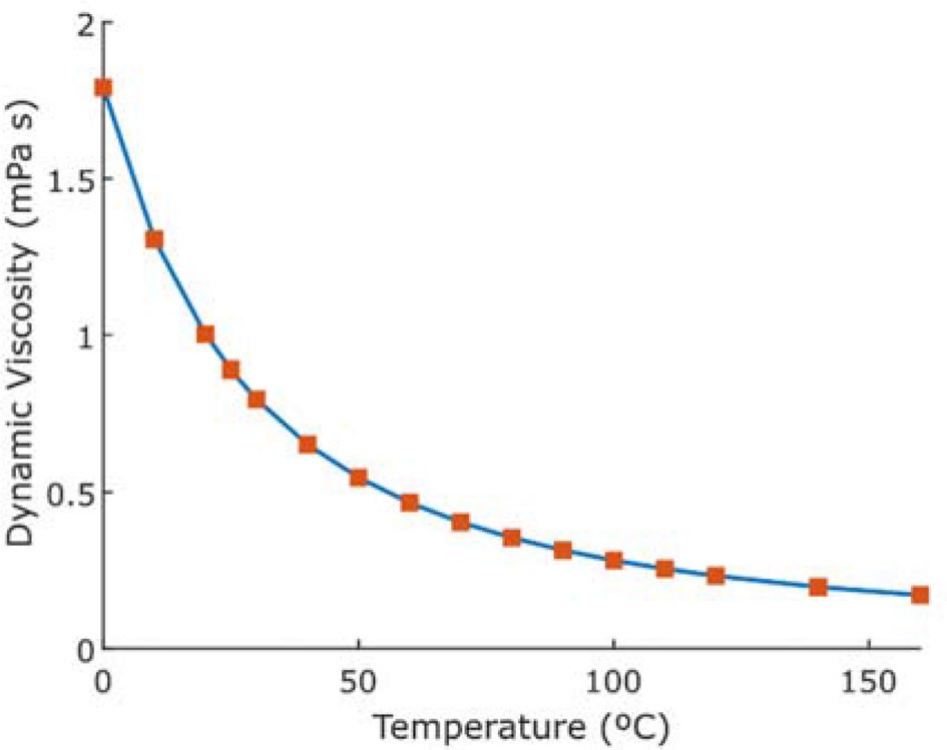
Temperature dependence of viscosity for saturated water^25^.

Herein, a fluctuation from the pump will change the pressure upstream of the stabiliser, while the downstream pressure is kept constant by adjusting the pressure drop through the stabiliser. Increasing the temperature in the restrictor will decrease the viscosity, Fig. 1, which increase the flow rate and lower the pressure drop, Eq. (1).

Decreasing the temperature will, on the contrary, raise the restriction and detain the flow. In this way, the use of the buffer capacitance, *V*_*bc*_, is not tied to the volume of the stabiliser, but to its ability to change the compression of the fluid^25^.

2 $${V}_{bc}=\beta {V}_{upstream}\frac{d{P}_{upstream}}{dt}$$

In the equation, *V*_*upstream*_ is the total volume upstream of the stabiliser including the pump volume, while *β* is the compressibility of the fluid. The change of upstream pressure, *dP*_*upstream*_, over time, is caused by fluctuations from the pump and from the heater changing the viscosity of the fluid in the stabiliser. If a fluctuation causes the initial flow rate, *Q*_*1*_, to temporary decrease to *Q*_*2*_, the stabiliser will compensate for this by increasing the temperature and changing the viscosity of the fluid in the stabiliser from *µ*_*1*_ to *µ*_*2*_, resulting in a pressure change upstreams of

3 $$\frac{d{P}_{upstream}}{dt}={P}_{t0}\left(1-\frac{{Q}_{2}}{{Q}_{1}}\times \frac{{(P}_{downstream}+\Delta {P\left({\mu }_{2}\right)}_{stabilisator})}{{(P}_{downstream}+\Delta {P({\mu }_{1})}_{stabilisator})}\right)$$

Whether a fluctuation can be fully damped depends on its magnitude and frequency in relation to the buffer capacitance. A fluctuation causing a difference of *dQ* for a certain time *t*, requires a buffer capacitance of

4 $${V}_{bc}=dQ\times t$$

## Materials and methods

### Stabiliser chip

The stabilisers were constructed from two bonded 1.1 mm thick borosilicate 4-in. wafers and had outer dimensions of 6 × 8 × 2.2 mm. A 10 mm long restrictor channel was wet etched with concentrated HF to a depth of 26 µm, corresponding to a channel volume of 16 nL. The inlet and outlet were etched to 70 µm with an addition to the total volume of approximately 7 nL each, depending on how far in the connecting capillary and epoxy glue went.

Heaters and temperature sensors were integrated by sputtering 110 nm Pt on top of a 30 nm adhesion layer of Ta. The thin films were embedded in 150 nm deep trenches and patterned with a lift-off process. Thermal wafer bonding was performed before the bonded wafers were diced into chips. A detailed description of the assembling of the flow and the electrical connections to the chip as well as the full fabrication description can be found in Supplementary Information.

### Experimental setup

An assembled chip was connected between two pressure sensors at 400 Hz (33X, Keller) with a flow sensor at 3 Hz (SLG1430-480, Sensirion) downstream the chip. The pressure sensors have a precision of 0.01% F.S. and an accuracy of 0.05% F.S. (30 mbar and 150 mbar, respectively), and the flow sensor has an accuracy of ± 10% of reading. Pressure or flow stabilisation was performed by adjusting the heat with a power supply (QL355TP, TTi), based on external feedback signals from the downstream sensors. The resolution of the stabilisation is limited by the feedback sensor resolution and by the power supply resolution. The time resolution varied between experiments but was typically around 1.4 s, limited by the computer quality and chosen loop pause. The assembled chip was placed on a water-cooled table with a cooling paste in between. The cooling water temperature was set to 8 °C, for us the lowest possible without developing condense between connections.

**Figure 2:**
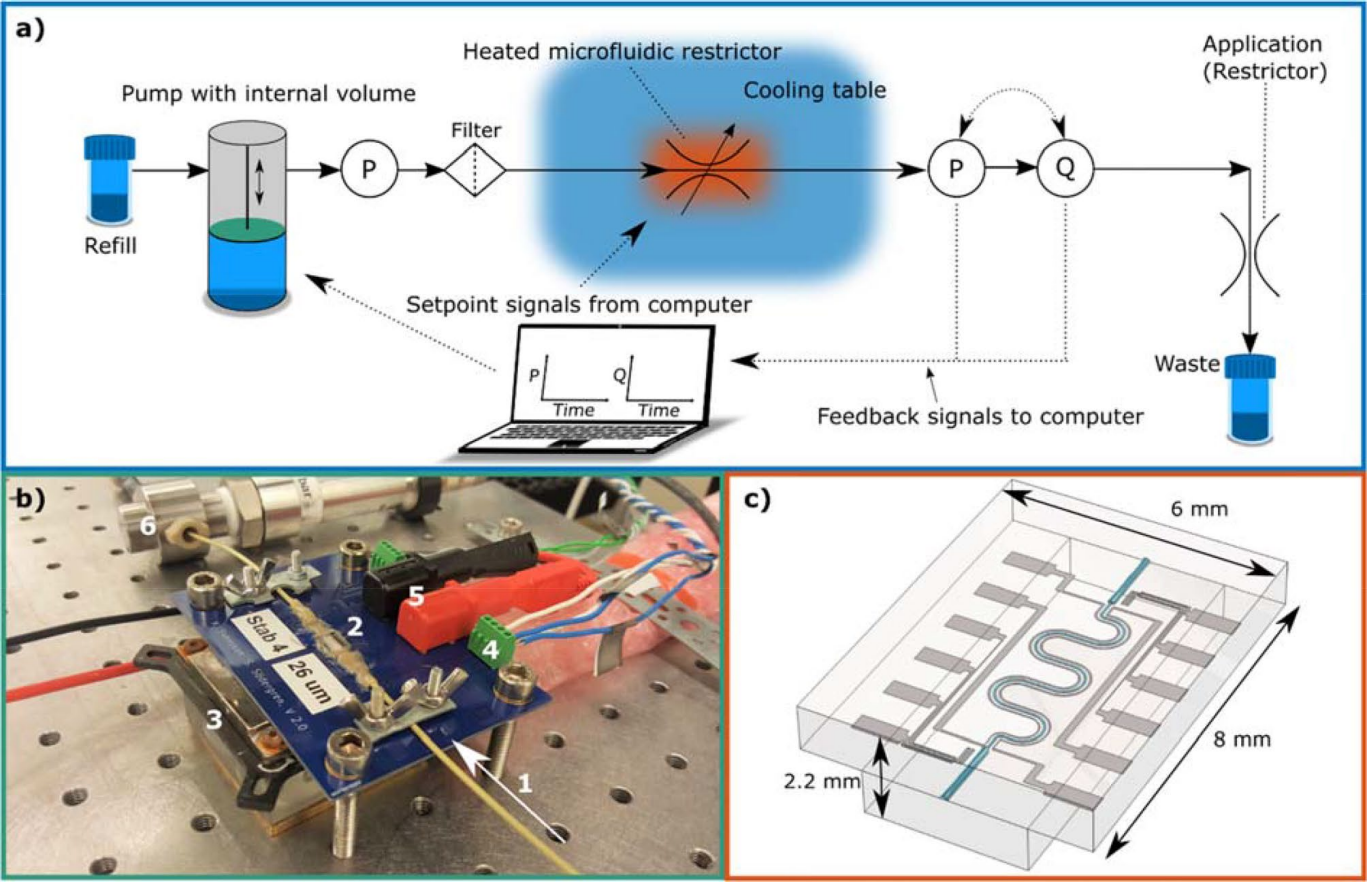
Images showing the experimental setup and the microfluidic chip used for stabilisation. (**a**) Schematic illustration of the flow system and the control system where P is the pressure sensors and Q is the flow sensor. (**b**) A photo of the stabiliser in the experimental setup; 1. Inflow through PEEK tubing, 2. microfluidic chip, 3. cooling table, 4. temperature sensor connectors, 5. heater connectors, 6. pressure sensor. (**c**) A 3D illustration of the chip with the flow channel in turquoise, the integrated thin films in grey, and the glass as semi-transparent.

To reduce clogging, a filter of 2 µm (A-702, IDEX) was placed upstream the stabiliser and, to simulate an application with a pressure drop, a restrictor capillary was placed at the end of the flow system. Three different pumps were evaluated; an HPLC pump (model 515, Waters), an ISCO pump (100 DM, ISCO Teledyne), and a Harvard syringe pump (PHD 2000 Infusion, Harvard). In the case of the Harvard syringe pump, a 10 mL glass syringe was used (Gastight #1010, Hamilton). Deionised water was degassed with ultrasonic sound for 30 min before use. A flow scheme of the experimental system can be found in Fig. 2, together with a photo of the setup and a 3D illustration of the chip.

### Measurements and PID control

In line with the theory, the downstream pressure is increasing when the temperature in the stabiliser is increased, and decreasing for the opposite. This was used to control the voltage of the power supply via a feedback loop from either the pressure or flow sensor, placed downstream of the stabiliser. A setpoint of the desired pressure or flow rate was selected and the error between the setpoint and the actual value was calculated. The PID parameters were manually set and tuned for different flow rates and different chips, as the resistance of the heater and the pressure drop varied. An initial effect of 0.15–0.2 W was applied pre-experiment to enable regulation in both directions.

To demonstrate the stabilisation concept, the set flow rate of the ISCO pump was alternated between 75 and 85 µL/min every second minute. Meanwhile, a pressure corresponding to 80 µL/min was set to be maintained by the stabiliser chip. For comparison, the same experiment was performed with the chip in passive mode with a constant power of 0.32 W. The pump volume was below 10 mL during the active experiment and within 15 and 12 mL during the passive experiment. The experiment with active stabilisation was also repeated with a smaller pump volume of 4 mL, demonstrating a condition where the buffer capacitance is not sufficient.

To make a quality validation of the stabilising performance, a comparable study was made using three different pumps (HPLC, ISCO, and Harvard). The pressure was measured over time while the stabiliser was either active, passive, or disconnected. The setpoint for the active stabilisation was taken from the mean value of the pressure without regulation. Precision and accuracy were calculated with values from the time the desired stabilised value was achieved, generally five minutes into each run. In this study, precision is expressed in relative standard deviation (RSD).

If the pump flow has a constant offset from the set value, the stabilisation can only maintain the value for so long. To extend the applicability and compensate for this, one more parameter was added to the regulation. For the computer-controlled ISCO pump, this parameter was the pump setting of either the pressure or the flow rate.

In these cases, a preferred span of the voltage for the heating was set to 13–17 V. If the voltage crossed the limits, a PI regulation of the pump setting started, based on the error between the mean value of the span and the actual value of the voltage. A voltage below the span caused the regulation to decrease the pump setting, forcing the voltage to increase to maintain the flow, while the opposite applies for a voltage above the span. Except for the error, the slope of the voltage was also considered. An ever-increasing value indicates that the maximum effect will eventually be reached, with a drop in downstream pressure as a result. To prevent this, the pump setting was increased for a positive slope and decreased for a negative slope.

In the case of pressure stabilisation, the setpoint was set to 15 bar, and measurements were made by controlling either the flow rate or the pressure of the pump. In the case of flow stabilisation, the flow sensor was placed closest to the stabiliser for quicker feedback, Fig. 2a. The flow rate was stabilised at either 5 µL/min or 30 µL/min and both measurements were made by controlling the pump flow rate.

For manual pumps, using data from the voltage regulation, the settings could be manually adjusted if an offset was detected. But, in the same manner as changing the pump setting, the setpoint can instead be adjusted to better fit the actual value. This gives a more accurate value of the flow while also stabilising it. To demonstrate a manual pump the ISCO pump was used without computer control. For pressure stabilisation, the pump flow rate was constantly 100 µL/min and the initial setpoint was 17 bar. For flow stabilisation, the pump flow rate was constantly 40 µL/min and the initial setpoint was 35 µL/min.

## Results

### Stabilisation concept

**Figure 3:**
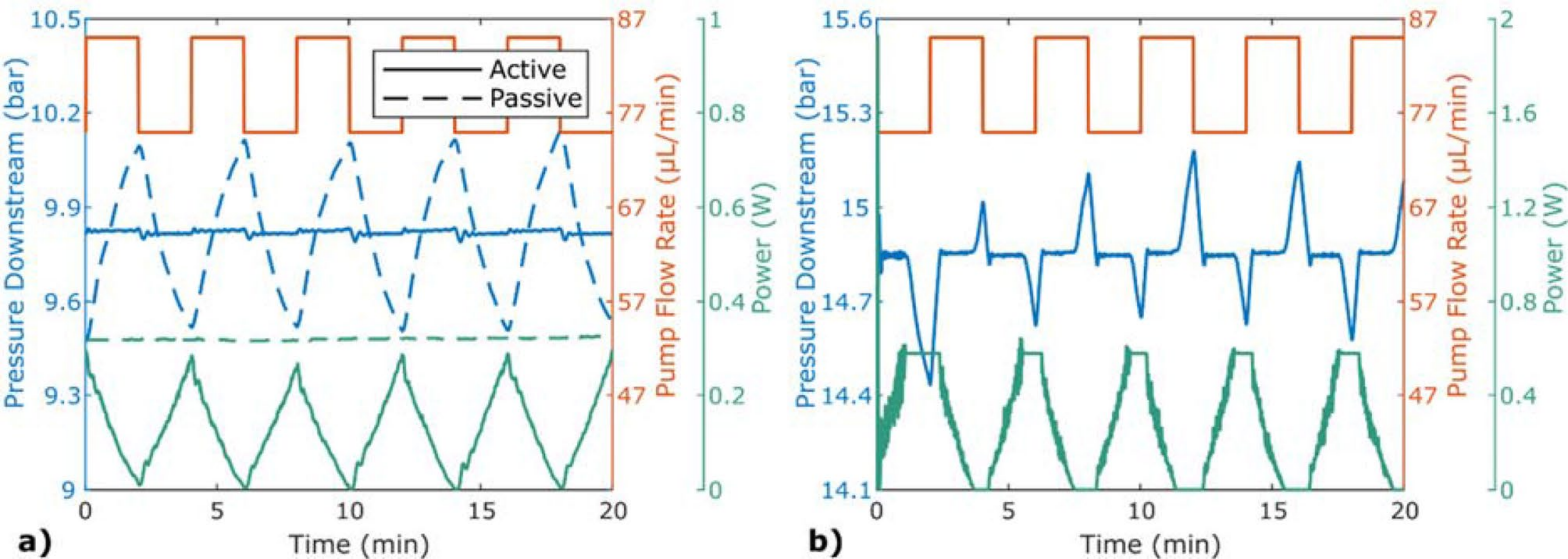
Pressure stabilisation with the set flow rate altering between 75 and 85 μL/min every second minute. The experiments were performed using an ISCO pump. (**a**) Active stabilisation (solid lines) was performed by PID-regulating the heater power of the stabiliser using an external pressure sensor downstream of the stabiliser as a feedback sensor. Passive stabilisation (dashed lines) was performed using constant power. During the passive run, the pump volume started with 15 mL and during the active run, the pump volume started at 9 ml. (**b**) The experiment was repeated with a smaller initial pump volume of 4 mL, to show that if the buffer capacity is too small, the stabilisation will not last long.

Results from pressure stabilisation with an alternating set flow rate from the pump can be studied in Fig. 3. In (a), the pressure downstream the chip was kept at a mean value of 9.82 bar ± 0.29% and, to keep the pressure stable, the power altered between 0 and 0.29 W. In contrast, for the passive stabiliser with a constant power of 0.32 W, the pressure was alternating with the alternating flow rate. In (b), the experiment was repeated with less volume in the pump and the pressure could not be maintained for each alternating cycle. When the flow rate was lowered and the power reached its set maximum value of 0.58 W, the buffer fluid was depleted and the pressure decreased. Accordingly, for a higher flow rate, the buffer was filled when the power reached zero, making the pressure rise. The difference in absolute pressure between the two figures was caused by a change of the downstream restriction.

The temperature in the stabiliser rises with increasing power, a simulation found in Supplementary Information indicates that a power of 0.4 W corresponds to a maximum of 70 °C at a flow rate of 100 µL/min. However, the pressure sensors upstream and downstream of the chip did not experience any temperature changes, apart from sub-degree fluctuations of the room temperature. This indicates that the heated volume leaving the stabiliser is small enough to return to its original temperature and not affect the rest of the system.

### Quality validation

**Figure 4:**
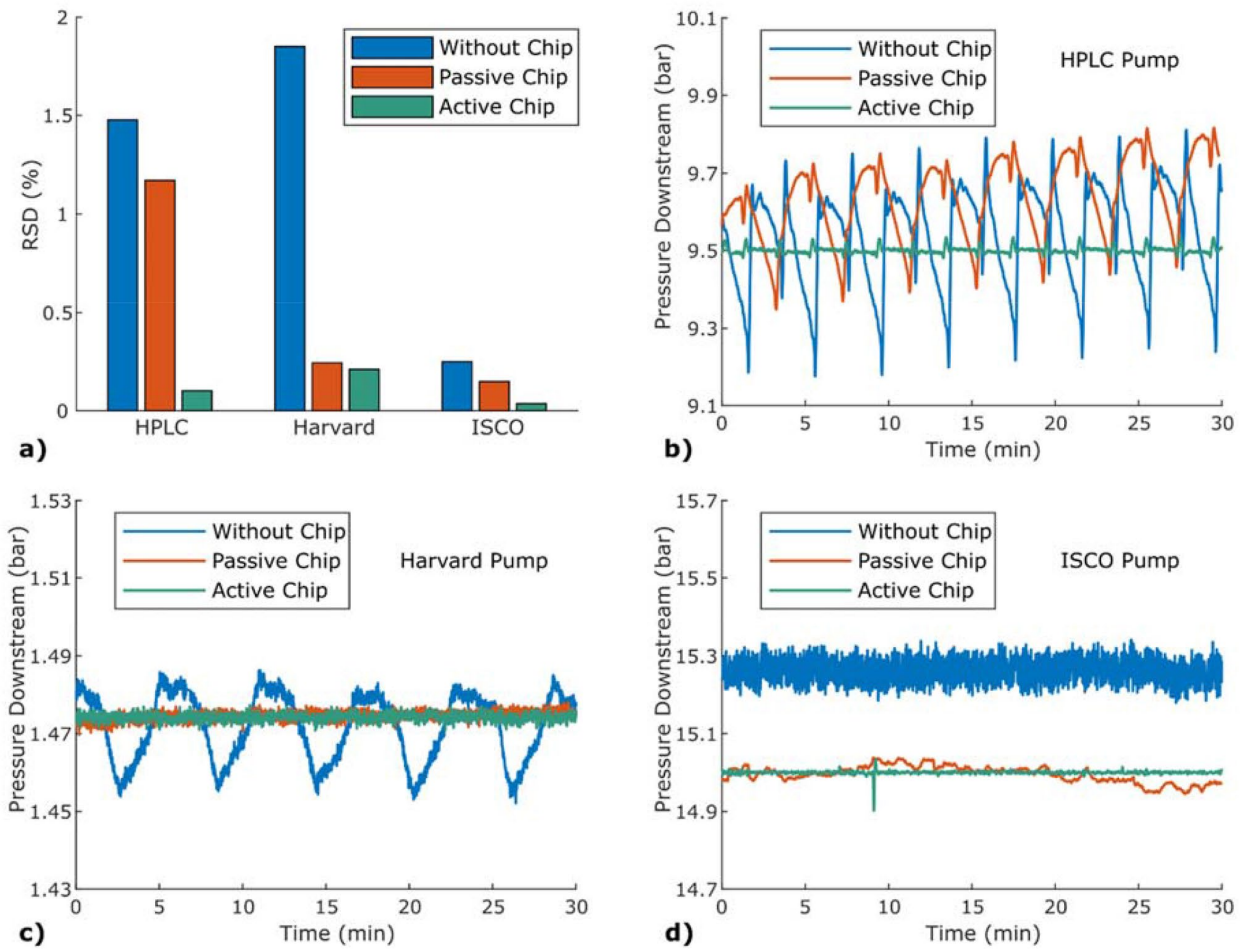
Pressure measurements for three different pumps with the stabiliser passive, active, or disconnected. (**a**) A bar plot showing the relative precision of the downstream pressure. Note that the relative precision is affected by the absolute pressure, which differs between the pumps. (**b**) HPLC pump with a set flow rate of 50 μL/min. (**c**) Harvard pump with a set flow rate of 30 μL/min. (**d**) ISCO pump with a set pressure of 15 bar for the measurement without stabilising chip, 45 bar for the passive chip and 42 bar for the active chip.

The precision and accuracy were improved when the stabiliser was connected, both passive and active, Fig. 4. The best relative precision of 0.035%, and accuracy of 8.0 ppm (4.9 mbar and 0.11 mbar, respectively), were achieved using the ISCO pump. For the Harvard pump, the pressure drop through the passive chip was 7 bar, which reduced most of the fluctuations. However, there are still some longer term fluctuations with lower amplitude, which are reduced with an active chip. A zoomed in and smoothed figure of this can be found in Supplementary Information. The HPLC pump had a pressure drop through the passive chip of 11 bar, while the resulting flow rate from the ISCO pump was around 100 µL/min.

### Applicability demonstration

**Figure 5:**
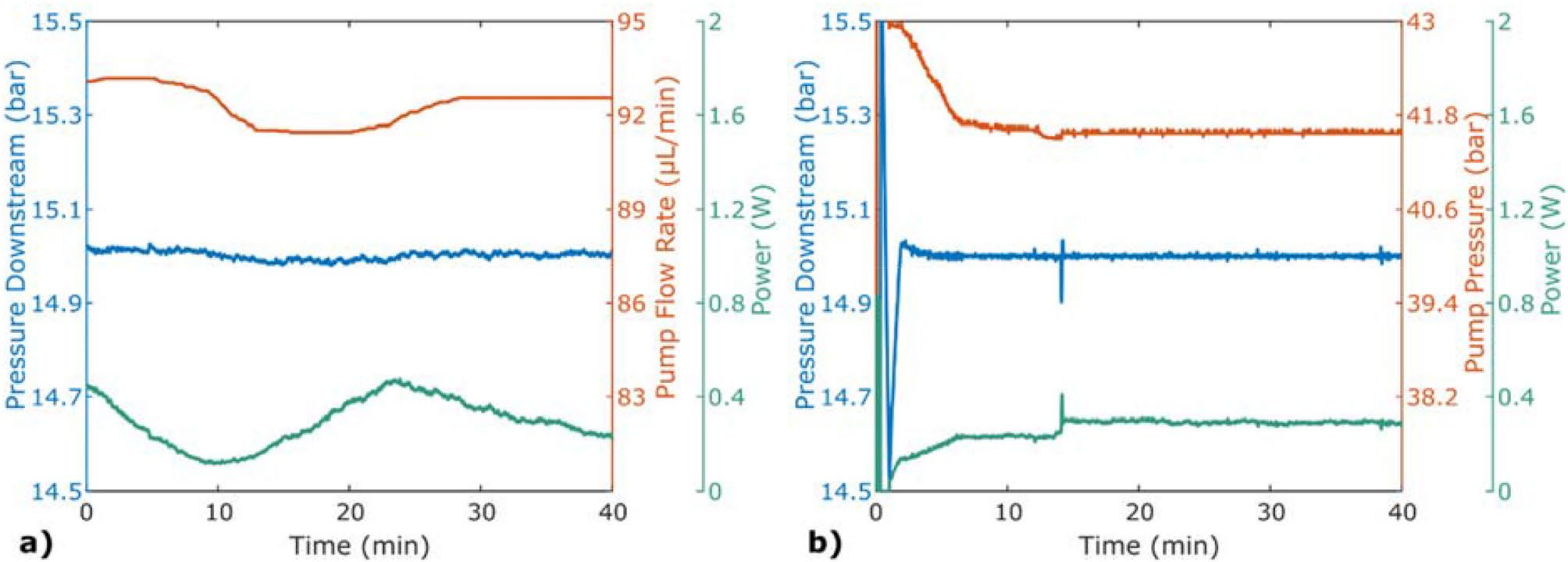
Stabilisation of pressure at 15 bar using an actively heated microfluidic chip, plotted against the control parameters. In the experiment, an ISCO pump was used with an external pressure sensor for feedback. The control parameters are the heater power and (**a**) pump flow rate, or (**b**) pump pressure.

To enable stabilisation of pressure using a pump with only flow rate control, the set flow rate of the pump was included in the regulation, Fig. 5a. When the power is getting critically low, the flow rate of the pump is decreased, and when the power is about to reach its maximum, the flow rate is increased, to avoid this. Here, the relative precision was 0.065% with an accuracy of 0.014% (9.1 mbar and 2.0 mbar, respectively).

If the pump has pressure control, this can likewise be integrated into the regulation, Fig. 5b. As seen in Fig. 4d, the pump had a pressure offset of 0.3 bar. However, with an active stabiliser, the relative precision was 0.035% with an accuracy of 8.0 ppm (4.9 mbar and 0.11 mbar, respectively).

**Figure 6:**
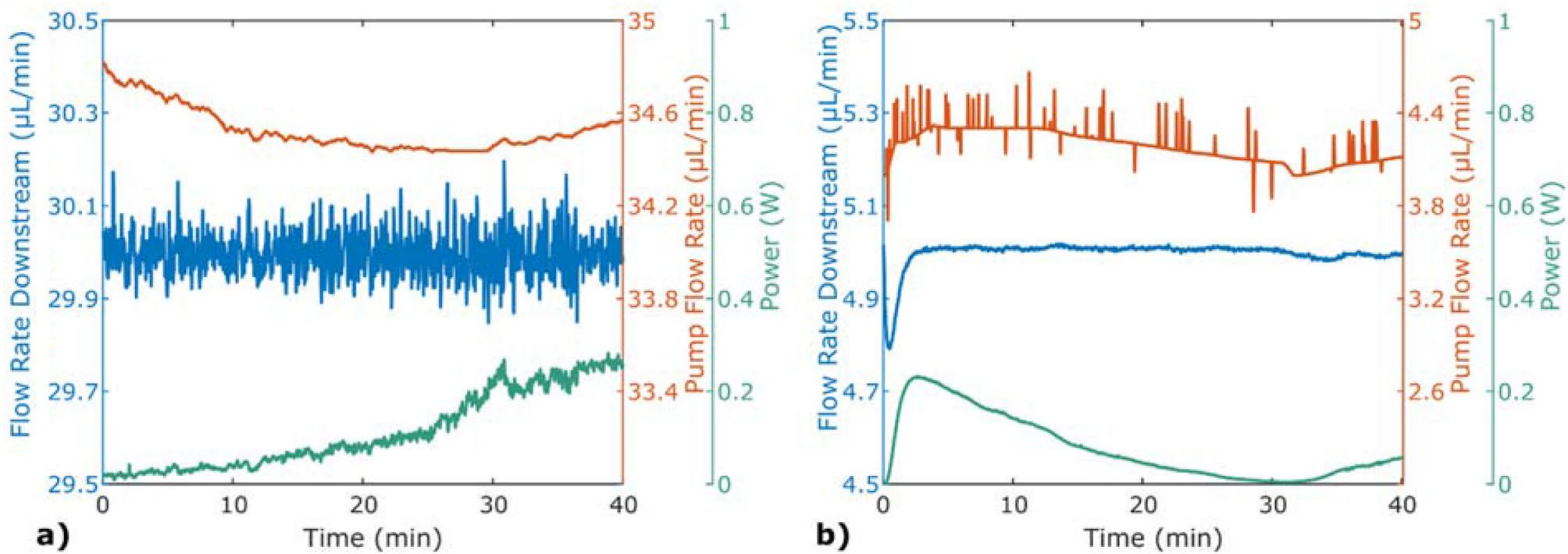
Stabilisation of flow rate, using an actively heated microfluidic chip, plotted against the control parameters. In both experiments, an ISCO pump was used with an external flow sensor for feedback. Stabilisation was performed for (**a**) 30 μL/min and (**b**) 5 μL/min.

To stabilise the flow rate, the flow sensor was used in the feedback loop instead of the pressure sensor. In Fig. 6, stabilisation of two different flow rates is presented. Both were performed by adjusting the flow rate of the pump and the power in the stabiliser. In (a), the relative precision was 0.14% with an accuracy of 0.011% (42 nL/min and 3.3 nL/min, respectively). In (b), the relative precision was 0.14% with an accuracy of 0.086% (7.0 nL/min and 4.3 nL/min, respectively). 

**Figure 7:**
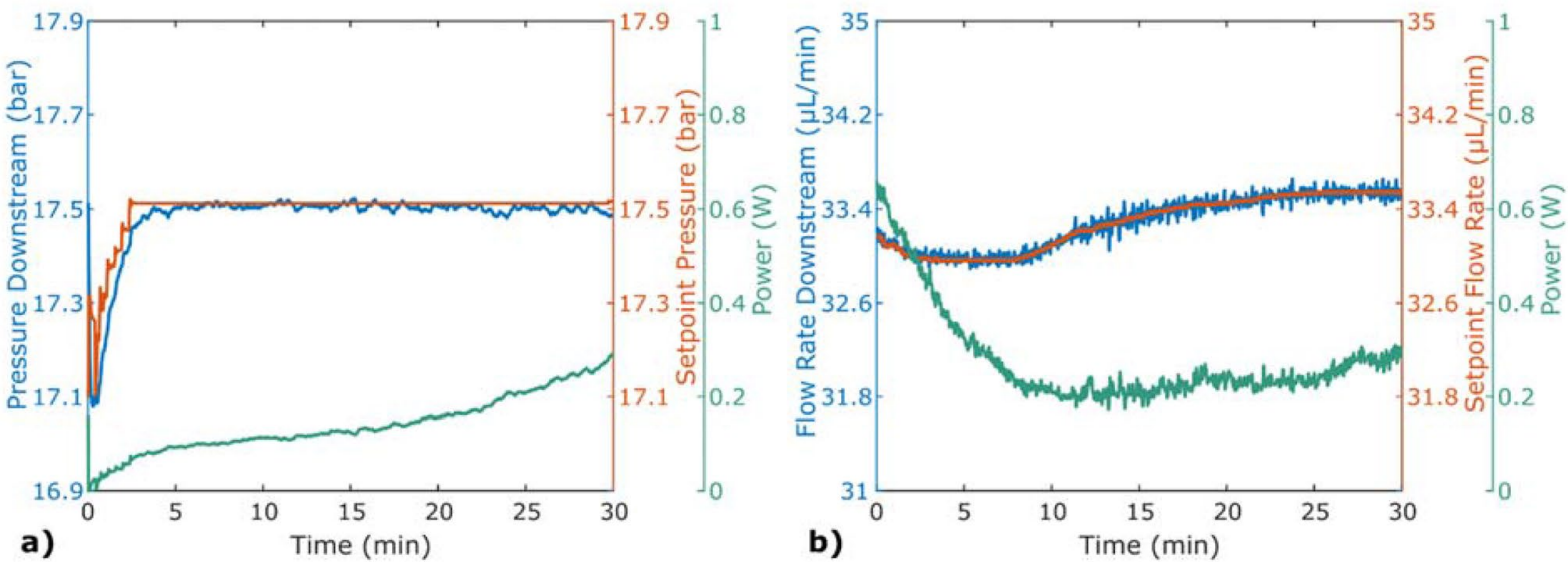
Pressure and flow stabilisation for a regulated setpoint. An ISCO pump was used in manual mode to demonstrate stabilisation for pumps that cannot be computer controlled. (**a**) Pressure stabilisation was performed with a pump set flow of 100 μL/min resulting in a downstream pressure around 17.5 bar. (**b**) Flow rate stabilisation was performed with a pump set flow at 40 μL/min, resulting in a flow rate around 33.5 μL/min.

Stabilising pressure or flow using a non-computer controllable pump, such as the Harvard or HPLC pumps, required another approach, Fig. 7. Here, the setpoint of the pressure or flow rate was regulated for stabilisation at the actual level provided by the pump. In (a), the relative precision was 0.048% (8.4 mbar) and in (b) the relative precision was 0.67% (220 nL/min).

## Discussion

In this work, it has been shown that the three evaluated pumps suffered from noise, fluctuations, and offsets. These are common issues that should be addressed, as an incorrect or fluctuating value can prohibit reproducible and reliable results. The HPLC pump often runs with a higher flow rate than used in this work, which reduces its fluctuations. However, pumps are expensive, making it beneficial to use the stabiliser to expand their working range.

A miniaturised stabiliser enables temperature actuation as the distances are short and the small volume reduces the required heat energy for temperature increase. Since only a fraction of the fluid gets warm, it also cools down quickly as it leaves the stabiliser, or when the power turns off. The stable and unaffected temperature in the pressure sensors also proves the applicability for temperature sensitive experiments. An experiment or sample injection downstream of the stabiliser will not experience any heating or cooling. Therefore, the temperature only has to be considered if the fluid from the pump is temperature sensitive. However, with a flow rate of e.g. 50 µL/min, the fluid will only be in the heated channel for approximately 19 ms.

The results shown in Fig. 3 demonstrate the concept of the presented stabilisation method. In (a), one can observe that a negative offset from the pump makes the stabiliser increase its power to maintain the pressure downstream. As the upstream pressure constantly decreases, the temperature needs to constantly rise. The upstream pressure is affected by the lowered flow rate and the temperature needed to access the buffer capacitance. Note that the flow rates shown in the figure are set flow rates of the pump, and not measured values. Because of the compressibility, even a sudden change in the pump setting will result in the flow rate gradually changing along with the pressure. As can be seen for the passive run, the blue dashed line in (a), the pressure never reaches a plateau between the altering cycles, indicating that the flow rate never reaches the extrema.

The reason that the pressure can be maintained in Fig. 3a but not in (b) is because the pump volume was lower for the experiment shown in (b), resulting in a lower buffer capacitance. It can also be seen that the first negative pressure peak is deeper than the following. This can be explained by the flow rate changing from having a constant value, 80 µL/min, to the altering flow rate, beginning with a negative offset. For the first cycle, the initial flow rate, *Q*_1_ in Eq. (3), will be 80 µL/min, while for the second cycle it will be higher, resulting in a larger buffer capacitance.

In Fig. 4, the results demonstrate the benefits and limitations of the stabiliser in passive mode, where it operates as a restrictor, and in active mode. For the Harvard pump, most of the fluctuations is reduced using the passive chip, while the HPLC pump needs active stabilisation to get a significant improvement.

Whether a passive restrictor is enough to reduce fluctuations depends on the size of the fluctuation, the restriction and the volume of the system. For example, if a fluctuation causes the flow rate from the pump to increase by 10%, the pressure throughout the system must also increase by 10%, before the flow rate in the end equalises the flow rate from the pump. The use of a restrictor will increase the upstream pressure, which will also increase the absolute change this percentage corresponds to. Because of the compressibility of the fluid, the pressure changes gradually. How steep the gradient is depends on how much fluid needs to be added to compensate for the compression. This volume will be proportional to the compressibility of the fluid and the total volume of the system. If the fluctuations are short and shifting, the pressure change will not keep up and the fluctuations will be dampened. Here, the biggest difference between the pumps, explaining the results, are the volumes of the systems. The HPLC pump has a very low internal volume of 100 µL resulting in quick pressure changes, while the Harvard pump has the volume of the 10 mL syringe. The ISCO pump has an even larger volume, up to 100 mL, and the noise is significantly improved with the restrictor. However, the difference in RSD is not as clear due to smaller original fluctuations. Of course, only an active stabiliser may handle drift in the pump.

The active-stabilisation quality was varying slightly for the three different pumps. This is a result of the original fluctuations from the pumps and the absolute pressure levels. In Fig. 4a, the RSD for active stabilisation is higher for the measurements using the Harvard pump, compared to the other two pumps. The absolute deviation are similar between the Harvard pump and the ISCO pump, but the lower the absolute pressure is, the higher the RSD gets. In addition, the PID parameters play a part in the quality of the stabilisation. The sharp pressure peaks, seen in Fig. 4b, are caused by the two pistons in the HPLC pump and place great demands on the regulation. Here, the response time of the system and the speed of the feedback loops are crucial for the stabilisation. The response time is affected by the time constant of the heating and the position and dead volume of the sensors. The development of an internal pressure or flow sensor in-situ the stabiliser would help improving the response time.

The quality of the stabilisation is ultimately depending on the external sensor. For the pressure, the best measured precision and accuracy was 5.1 mbar and 0.14 mbar, respectively. This can be compared with the precision of the sensor of 30 mbar, and accuracy of 150 mbar. The measured values are well below the sensors, meaning that the best quality is more or less the quality of the sensor. Consequently, it is crucial to make precise and well-calibrated sensors for higher stabilisation quality.

If the pump has a constant offset, the setpoint cannot be maintained for long-term experiments, even with a stabiliser. The buffer capacitance will over time either be depleted or saturated, corresponding to the power reaching the upper limit or decreasing to zero. This was solved by adding the pump setting of either the flow rate or pressure to the regulation, Fig. 5, which also enabled pressure control for pumps lacking it. The exact settings required to maintain a desired pressure downstream can be difficult to obtain due to pump offsets and varying restrictions in the flow system and over the experiment. With this regulation, only an initial approximation is needed, which is automatically adjusted if the power is heading towards an extreme, indicating an improper setting.

The sudden peak in Fig. 5b is a result of the chosen PID parameters. In the time interval around 10 min, the voltage is only slightly above 13 V, which means that a small fluctuation from the pump can push the voltage below the wanted range, resulting in a decrease of the set pump pressure. This decrease was, however, too sharp, making also the downstream pressure decrease and the voltage to spike to compensate for this. This is seen in the figure as the power slightly decreases and then spikes. After the spike, the power lands on a higher level than before. As the temperature difference between the two levels was very small, the change in upstream pressure is too small to notice in the figure.

The results in Fig. 6 shows that the principle for this stabilisation method is the same for flow rate as for pressure. The measured values are noisier in (a) compared to (b) because the used flow sensor has an accuracy as a percentage of reading, and the flow rate in (b) is larger by a factor 6. In Figs. 6 and 7, one can observe that the flow rates of the pump differs from the measured flow rates downstream. The reason for this is probably that, for an unknown reason, the sensor or pump offset was shifted between the experiments.

Many pumps are manual or cannot be integrated with a control system, like the Harvard pump and the HPLC pump. Therefore, experiments were done regulating the setpoint instead of a pump setting, with the results shown in Fig. 7. The setpoint is initially an approximation, which is PI regulated when the voltage is about to get too high or too low, in the same manner that the pump setting was regulated in the previous experiment. It might seem odd to change the wanted setpoint, but, as this does not change the pump settings, the conditions are still the same. Performing stabilisation on the actual value with this method is a way to enable reproducible results. By doing this, both more stable and accurate values are achieved. If an offset is revealed, it is also possible to manually change the setting of the pump to achieve the actually wanted conditions.

## Conclusion

An active microfluidic pressure and flow stabiliser was presented. It has delivered a relative precision of 0.035% and an accuracy of 8.0 ppm versus the external pressure sensor. The device has a very small dead volume of 16 nL and can be integrated with µTAS systems. The applicability is wide due to the high-pressure resistance and the chemically inert borosilicate glass material. The device has been used for pressure and flow stabilisation, at higher and lower pressures and for commonly used pumps, such as ISCO and Harvard syringe pumps and HPLC piston pumps. By this paper, an increased fluid mechanic control has been achieved for high-pressure microfluidic applications like extraction, synthesis, and analysis.

## Supplementary Information

Supplementary Information.
