# Supplementary material for: Microfluidic active pressure and flow stabiliser
Source: Sci Rep. 2021 Nov 18;11:22504. doi: 10.1038/s41598-021-01865-4 (PMC8602347; doi:10.1038/s41598-021-01865-4)
Supplement: Supplementary file 1 — Supplementary Information. [file 41598_2021_1865_MOESM1_ESM.docx]

**Supplementary information**

Chip Fabrication

Each stabiliser chip was made out of two RCA cleaned 1.1 mm thick, circular, 4’’ borosilicate glass wafers (Borofloat 33, Schott). To form channels in the wafers, 250 nm of Mo was used as a hard wet etch mask. Mo was sputtered (CS730S, Von Ardenne) on one side of each wafer, and to reduce the density of pinholes and increase the adhesion, the wafers were thoroughly pre-cleaned. The cleaning was performed by first removing inorganic contaminants by immersing the wafers into a Phosphoric Acetic Nitric (PAN) solution for 20 min with a mixture of 180 H_3_PO_3_ : 11 HAc : 11 HNO_3_ : 15 H_2_O. Thereafter, the wafers were cleaned in an oxygen plasma at 1000 W for 20 min to remove organic contaminants and increase the adhesion to the Mo-film.

The channel structures were formed with a standard UV-lithography method. First, the Mo sputtered wafers were primed with an adhesion promoter primer, HMDS, by inserting them into a primer furnace (Star 2000, Star) for 30 min. They were thereafter deposited with 1 µm photoresist (1813, Shipley) by spinning the wafers at 6000 rpm for 30 s. The photoresist was soft baked at 110 °C for 75 s on a hotplate and then structured with UV-exposure. One lithography mask was used to pattern channel restrictions on one wafer, and a second mask was used to form the inlet channels on the other wafer. The wafers with the exposed photoresist were immersed into a developer for 45 s. After development, the wafers were rinsed in nitrogen bubbling DI-water for 1 min and blow-dried with nitrogen gas during a spin, a procedure that followed all following wet etch steps. To etch the Mo and expose the glass, the wafers were etched in the previously described PAN solution for 4 min. An isotropic wet etch of the glass was then made in 49 wt.% HF. After the glass etching, the photoresist was removed with ultrasonic acetone. Finally, the Mo was stripped with the named PAN solution.

Pt thin-films were added on the wafer with etched restriction channels. This was performed by first sputter Mo onto the wafer, on the same side as the channel, right after a 20 min oxygen plasma etch at 1000 W. The Mo is in this case mainly used as a lift-off layer, but also as a reflective substrate that increases the lithography resolution. Due to the etched channels on the wafer, a 4 µm thick spray resist was added onto the Mo thin-film using a spin-spray coater (101, EVG). This is a rather thin spray resist and was used to get a better lithography resolution compared to a thicker one. However, it gives an increased risk of non-covered edges and to avoid this, and get optimal edge coverage, the chuck was heated to 80 °C during the deposition. Soft baking was made on a hotplate at 90 °C for 3 min. The thin-film structure was formed with UV-lithography and the resist was developed for 3 min. The Mo was etched in the PAN solution for 4 min. Trenches for the thin-film was created in a buffered oxide etch (BOE 1:7, J.T.Baker). The thin films were made in a table-top sputter (Q300T D Plus, Quorum) by first sputter 30 nm Ta and then 110 nm Pt without breaking the vacuum in-between. Lift-off was made in ultrasonic acetone for 20 min and then the Mo was striped in the PAN solution.

The activation for bonding was made by first cleaning the wafers in the PAN solution for 20 min, followed by an oxygen plasma for 20 min. Then the wafers were activated in nitric acid at 80 °C for 15 min before the pre-bond was made in a bond-aligner (MA6/BA6, Karl Süss). The pre-bonded wafers were placed on a SiN substrate in a furnace (Micro TF-6, Koyo Lindberg) and were thermally bonded at 630 °C with a dwell at 6 h and a ramping rate of 1 °C min^-1^. As a final step, the bonded wafers were first diced (DAD 320, Disco) halfway through, to open up the electrical pads, and then diced into individual chips.

Chip assembly

To electrically contact the stabiliser chips, each chip was mounted on a tailored printed circuit board (PCB). Conductive glue (CW2400, CircuitWorks) was placed on the electrical pads under a stereo microscope and the chip was placed with the Pt pads towards the electrical pads on the PCB. The glue was cured in an oven at 110 °C for 30 min. 4 mm connectors (FCR7350, CLIFF) was soldered to the PCB to get access to the heating elements and screw terminals (1725672, Phoenix Contact) were soldered to get access to thermal measurements.

To get fluid access to the chip, glass capillaries with 40 µm inner diameter and 105 µm outer diameter (88224, Polymicro Technologies) were inserted into the inlet channels of the chip. The inlet channels were only etch to a depth of 70 µm to optimise pressure tolerance. Therefore, the capillaries were thermally pulled (P-1000, Sutter Instrument) in order to get a pointy tip that could fit into the inlets. The length of the capillaries were around 3-4 cm. The capillaries were then glued with a two-component epoxy (730, EPO-TEK) and cured in room temperature for 24 h. The reason for curing at room temperature is that the glue will flow too far into the channel and clog the capillary if an elevated curing temperature is chosen. The other end of the glued capillaries were inserted and glued into PEEK tubing (Upchurch, IDEX), and cured at 80 °C for 2 hours with the named epoxy. As a final step, the PEEK tubing on both sides of the chip were strain relieved using screws, nuts and cut out steel pieces.

Simulations


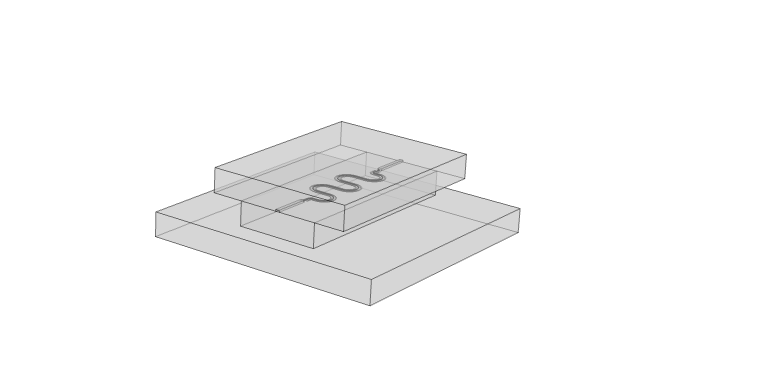
A simulation of the heating was made in COMSOL Multiphysics 5.6. The chip consist of a borosilicate glass-glass chip with water in the fluidic channel and platina electrodes on the sides of the channel. The chip is placed on an aluminum block, as seen in Figure 1, with a set temperature of 8 °C. The flow rate is set to 50 or 100 µL/min with the physic *Laminar flow*. The resistive heating is simulated with *Electric currents in shells* and added as *a Boundary heat source* in *Heat transfer in Solids and Liquids*. The power is 0.4 W. The multiphysics *Nonisothermal flow* is used to combine the physics. The resulting temperature at the glass-glass interface is shown in Figure 2.

**Figure 1.** (a) Geometry of the glass chip placed on a cooling block for the COMSOL simulation.


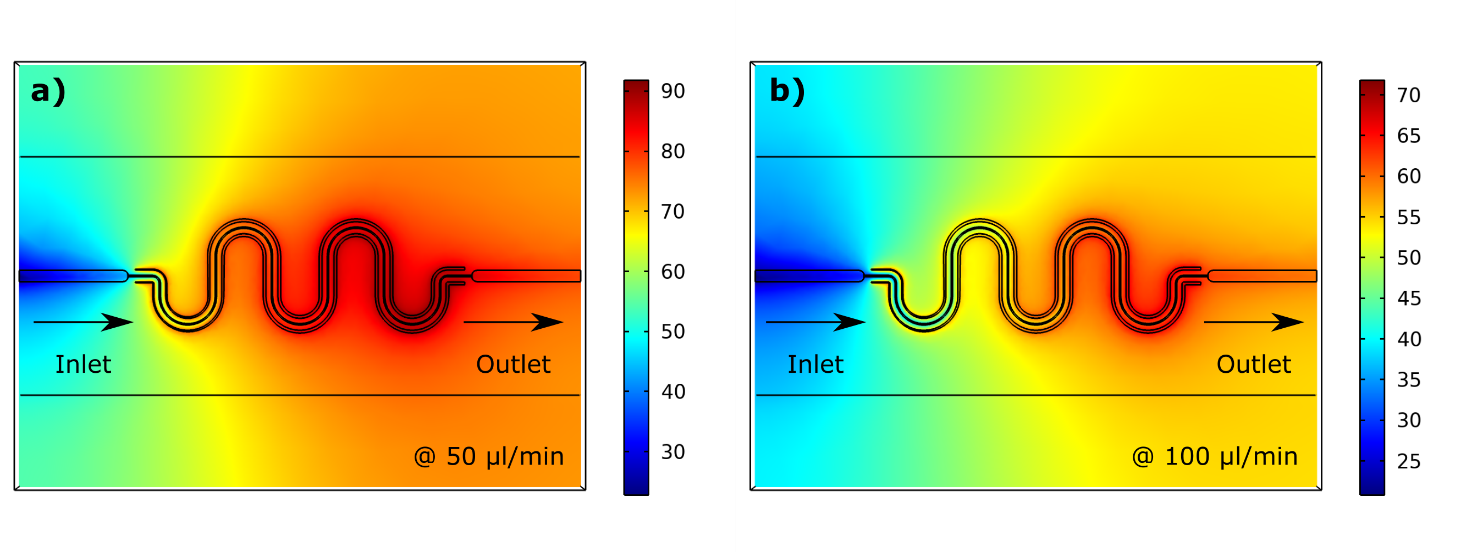


**Figure 2**. Resulting heat distribution at the glass-glass interface with a heating power of 0.4 W and a flow rate of (a) 50 µl/min and (b) 100 µl/min.

Results from quality validation


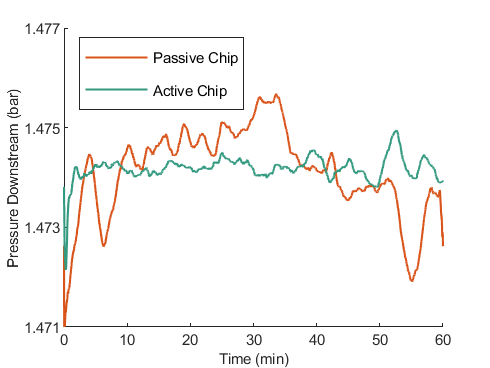


**Figure 3.** Measurement with the Harvard pump using a set flow rate of 30 µL/min with the stabiliser either passive or active. The data is smoothed with a moving average of 200 data point, corresponding to approximately 3 min.


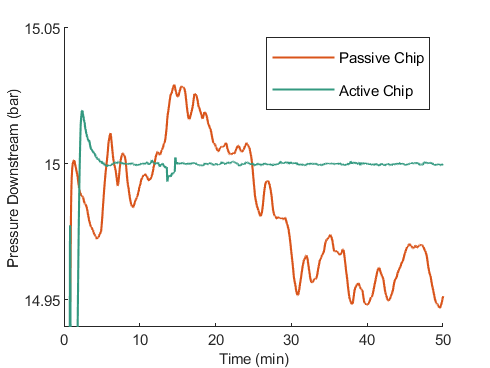


**Figure 4.** Measurements with the ISCO pump using a pump pressure of 45 bar for the passive chip and 42 bar for the active chip. The data is smoothed with a moving average of 50 data points, corresponding to approximately 1 min.
